# Assessment of patients waiting and service times in the ophthalmology clinic of a public tertiary hospital in Nigeria

**DOI:** 10.4314/gmj.v54i4.5

**Published:** 2020-12

**Authors:** Lateefat B Olokoba, Kabir A Durowade, Feyi G Adepoju, Abdulfatai B Olokoba

**Affiliations:** 1 Department of Ophthalmology, University of Ilorin Teaching Hospital, Ilorin, Kwara State, Nigeria; 2 Department of Community Medicine, Afe Babalola University, Ado-Ekiti, Nigeria; 3 Department of Medicine, University of Ilorin Teaching Hospital, Ilorin, Kwara State, Nigeria

**Keywords:** Service time, waiting time, ophthalmology clinic

## Abstract

**Introduction:**

Long waiting time in the out-patient clinic is a major cause of dissatisfaction in Eye care services. This study aimed to assess patients' waiting and service times in the out-patient Ophthalmology clinic of UITH.

**Methods:**

This was a descriptive cross-sectional study conducted in March and April 2019. A multi-staged sampling technique was used. A timing chart was used to record the time in and out of each service station. An experiencebased exit survey form was used to assess patients' experience at the clinic. The frequency and mean of variables were generated. Student t-test and Pearson's correlation were used to establish the association and relationship between the total clinic, service, waiting, and clinic arrival times. Ethical approval was granted by the Ethical Review Board of the UITH.

**Result:**

Two hundred and twenty-six patients were sampled. The mean total waiting time was 180.3± 84.3 minutes, while the mean total service time was 63.3±52.0 minutes. Patient's average total clinic time was 243.7±93.6 minutes. Patients' total clinic time was determined by the patients' clinic status and clinic arrival time. Majority of the patients (46.5%) described the time spent in the clinic as long but more than half (53.0%) expressed satisfaction at the total time spent at the clinic.

**Conclusion:**

Patients' clinic and waiting times were long, however, patients expressed satisfaction with the clinic times.

**Funding:**

Self-funded

## Introduction

Waiting time is the time patients spend in the clinic before being attended to by a health worker, and service time is the time spent receiving active services. Total clinic time is the total time spent by a patient in the clinic. It is the time difference between when the patient arrives the entrance door and exits the health facility.^1^ A major factor identified from a previous study as a cause of dissatisfaction in Eye care services is the long waiting time in outpatient clinics.^2^,^3^,^4^ Long waiting time translate to man-hour loss, and loss of income to the patients and their relatives. A survey of Eye care workers' opinion on ways to improve the cataract surgical rate in Kwara State, Nigeria has identified bad service delivery in terms of the patients waiting too long for doctors' consultation as a barrier to uptake of services. And also, that, the present patients protocol makes patients actually spend more time doing other things not related to the actual business they came for.^4^

In another survey sampling the opinion of the patients that use the Eye care services of the same tertiary hospital, majority still reported long waiting time particularly for doctors' consultation as the short coming in the services they received.^3^

The recommendation by the Institute of Medicine (IOM), United State of America is that, at least 90% of patients should be seen within 30 min of their scheduled appointment time.^5^ This is, however, not the case in most outpatient clinics in Nigeria. An average waiting time of about 173 min was found in Benin city, southern Nigeria,^6^ while in Ahmadu Bello University Teaching Hospital (ABUTH), Zaria, north-west Nigeria, a mean waiting time of 97.2 minutes was observed.^7^

Consequently, it is important that the Eye care providers start addressing the problem of long waiting times in outpatient clinics. Several measures have been suggested to address long waiting times such as electronic appointment, staggering of patients' appointments, increasing staff numbers etc.^8^ However, each clinic or facility has its peculiar problems, and it is best to start by investigating the existing patient flow in the Eye clinic before proposing solutions to the problem.^9^

This study assessed patients' waiting and service times in the out-patient general Ophthalmology clinic of the University of Ilorin Teaching Hospital (UITH), Ilorin. The objectives were to; determine how long patients spend at each of the service stations when presenting to the outpatient clinic, assess patients' opinion on the waiting and service times at the out-patient clinic and assess patients' feedbacks on how to improve their experience at the clinic.

## Methods

### Study area

The study was a descriptive cross-sectional study carried out over 14 clinic days in March and April 2019 at the Out-patient General Ophthalmology clinic of UITH, Ilorin. University of Ilorin Teaching Hospital is a referral hospital with 600 bed capacity. Ophthalmology department has eight Consultant Ophthalmologists, twenty resident doctors, two Optometrists, eight Ophthalmic Nurses, and other support staff. The department runs outpatient general Ophthalmology four days in a week (Monday, Wednesday, Thursday and Friday) and six other sub-specialty clinics. An average of 900 patients (both new and follow up) pass through the out-patient general clinic every month.

### Inclusion criteria

All patients, 18 years and above presenting to the Outpatient General Ophthalmology clinic of UITH.

### Exclusion criteria

1. Emergency patients who needed urgent attention
2. Hospital staff as responses may introduce bias
3. Children and patients younger than 18 years of age.
4. Those that declined consent

### Sample size estimation

The minimum sample size was calculated using the formula:

n = Z^2^pq/d^2^

n = sample size,

Z = standard normal deviate at 95% confidence level =1.96

P = 0.79 (79%).^10^

n∼ 255

With a study population less than 10,000, the formula N_f_ = n/1+ (n/N) was used to estimate the required sample size.

N = Total number of patients seen per day in the clinic (55) × Total number of days of the study (14 days)

N = 770. Thus,

N_f_ ∼ 192 patients.

Accounting for non-response rate of 10% of the sample size; the calculated sample size is ∼ **212**

### Sampling technique

A multi-stage sampling technique was used to select patients for the study. Stage 1 involved categorization of the patients into two Strata of old and new patients. Stage 2 involved selection of the respondents. Systematic sampling technique was used to select the required number of respondents from each group. The calculated sampling interval was 4, thus for both old and new patients, the first respondent for each day was selected from among the first four using simple random sampling by balloting; and thereafter subsequent respondents were selected using a sampling interval of four.

### Data collection

Data collection was with the use of a timing chart and a self-administered questionnaire. The timing chart had the patient's biodata, arrival time, time in and out of each service station. (Appendix 1). The questionnaire is an experience-based survey card to assess patients experience at the clinic and proffer ways to improve their experience.

### Pre-test/ reliability and validity of research instrument

The timing chart and questionnaire used were validated at the out-patient Ophthalmology clinic of General Hospital, Ilorin. The hospital offers secondary Eye care services with similar spectrum of patients.

A total of 23 participants (10% of proposed sample size) were randomly selected for the pre-test and tests of reliability. The inter-rater and the test-retest methods was employed to measure the reliability of the timing chart and questionnaire respectively. The inter-rater observers' scores and those of the subjects in the test-retest were correlated and an alpha Cronbach value of 0.83 was gotten. Face and content validity of the tool was also ensured by a team of panellists vast in this area of research who examined the tools.

### Study procedure

Trained Research Assistants were stationed at all service points at the clinic. The Research Assistants arrived at the clinic at 6am and waited at the clinic entrance to record the arrival time of the patients. The first patient was recruited by selecting from among the first four patients that arrived at the clinic by balloting. Thereafter, every 4^th^ patient in a systematic sampling was recruited until the required sample size is completed. The same selection process was applied to the new patients and the follow up patients separately. Informed and written consent was taken from the patients.

Those that consented were giving a timing chart to be taken along to each service station (s) he/she attended. The time in and out of each station was filled by the Research assistant at every station. The completed timing chart was collected, and departure time noted on the chart by the Research assistant at the Record room after the patients were booked for his/her next appointment. Each patient was then given a questionnaire to assess their experience at the clinic and proffer ways to improve their experience.

### Ethical approval

Written informed consent was taken from all the patients recruited for the study. The quality of care given to the patients was not affected by their decision to or not to participate in the study. Ethical approval for the study was obtained from the Ethical Review Board of UITH (ERC PAN/2019/04/1891) on 27/03/2019

### Data analysis

The data generated was analysed using statistical package for social sciences (SPSS) version 24 and Microsoft Excel 2013. Univariate analysis was carried out to determine the frequency distribution of the socio-demographic variables and other parameters of the study. Frequency, percentage and mean/median of variables were generated.

Patients' total clinic time was cross tabulated with patients' socio-demographic parameters, arrival time and clinic days to check for association. Pearson's Chi square test was used to establish such association. The mean difference between waiting time at each service station and the total waiting time was obtained using Student t-test. The mean difference between service time at each service station and the total service time was also obtained using student t- test. Pearson's correlation was done to establish the relationship between patient's total clinic time, service time, waiting time and clinic arrival time. The level of statistical significance was taken as p < 0.05.

## Results

A total of 226 patients were recruited across all the clinic days. The patients were both new patients (63, 27.9%) and follow-up patients (163, 72.1%). The mean age of the patients was 47± 19.5 years (range 18 – 89 years). Female to male ratio was 1:0.9 (Table 1)

**Table 1:** Socio-demographic characteristics of the patients

Socio-demographics characteristics	Frequency n (%)
**Gender**	
**Female**	116 (51.3)
**Male**	110 (48.7)
**Age(years)**	
**≤ 30**	68 (30.1)
**31 to 60**	92 (40.7)
**> 60**	66 (29.2)
**Education**	
**None**	51 (22.6)
**Primary**	25 (11.0)
**Secondary**	40 (17.7)
**Tertiary**	110 (48.7)
**Occupation**	
**Civil servant**	54 (23.9)
**Artisan**	8 (3.5)
**Farmer**	11 (4.9)
**Retired**	23 (10.2)
**Student**	63 (27.9)
**Trader**	67 (29.6)

**Figure 1:**
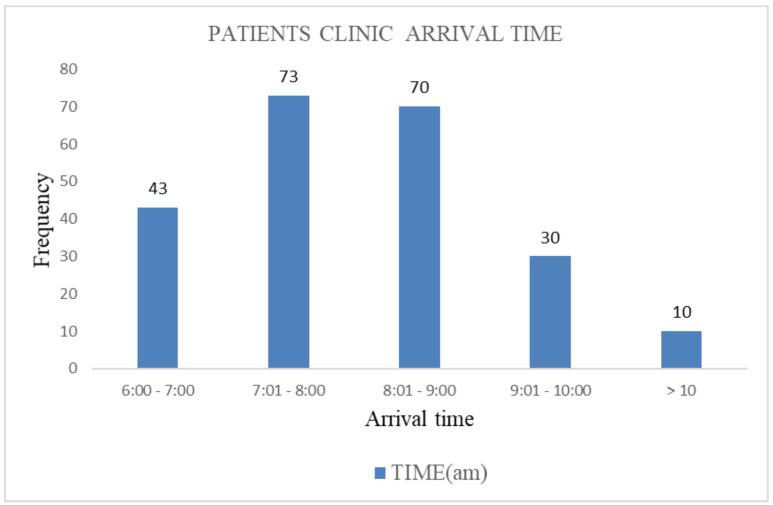
Bar chart showing the patients' clinic arrival time

Figure 1 shows the mean patients' arrival time to the clinic was 8:04±1:03 am. All the patients arrived between 6:00 am and 11:50 am. Forty-three (19.0%) patients arrived the clinic before the clinic doors were opened at 7.00am. The majority of patients (32.3%) arrived the clinic between 7:01am and 8:00am.

More than half (53%) of the patients expressed satisfaction at the total time they spent at the clinic. Table 2 shows the total time spent by a patient in the clinic is not associated with the age, gender, employment status, educational level or the clinic day of the patients. The patients' arrival time at the clinic and patients' category are associated with the total clinic time.

**Table 2:** Factors associated with patients' Total clinic time

Variable	Total Time (Minutes)		χ^2^	p-value
	≤120(%)	>120 (%)		
**Age (years)**				
**<60**	17 (11.7)	128 (88.3)		
**≥60**	4 (4.9)	77 (95.1)	2.86	0.101*
**Sex**				
**Male**	12 (10.9)	98 (89.1)		
**Female**	9 (7.8)	107 (92.2)	0.65	0.415
**Occupational Status**				
**Employed**	12 (8.6)	128 (91.4)		
**Unemployed**	9 (10.5)	7 (89.5)	0.23	0.634
**Educational Status**				
**No formal education**	5 (9.8)	46 (90.2)		
**Formal education**	6 (9.1)	159 (90.9)	0.02	0.886
**Clinic Day**				
**Mon. & Wed.**	9 (9.2)	89 (90.8)		
**Thur. & Fri.**	12 (12.3)	116 (90.6)	0.00	0.961
**Patient Category**				
**New**	1 (1.6)	62 (98.4)		
**Old**	20 (12.3)	143 (87.7)	6.15	0.010*
**Arrival Time**				
**At/Before 8am**	4 (3.4)	112 (96.6)		
**After 8am**	17 (15.5)	93 (84.5)	9.66	0.002*

Table 3 shows one hundred and sixty-nine patients (75%) gave at least one feedback on how to improve the clinic.

**Table 3:** Patients' feedbacks on how to improve clinic experience

*Patients' feedback	n (%)
**Electricity supply**	80(47.3)
**Strict adherent to the first come first served rule**	52(30.8)
**Employ more doctors and nurses**	33(19.5)
**Improve work ethics of personnel (time wasting, early resumption)**	10(5.9)
**Reduce the cost of Services**	10(5.9)
**Purchase more equipment to minimise equipment sharing**	7(4.1)
**Provision of more seats**	7(4.1)
**Proper clinic direction**	5(3.0)

* Multiple responses

The feedbacks were grouped under three major categories: Electricity supply, personnel and equipment, and clinic organisation. The most frequently cited feedback is the need to provide steady electricity.

## Discussion

The sampled population was a mixture of both old and new patients and is reflective of the ratio of old and new patients attended to in the out-patient general Ophthalmology clinic per day (ratio 2.6 old: 1 new). The patients were recruited on all clinic days eliminating bias that could result from recruiting only on certain days of the week.

The mean age of patients in this study was 47.0 years. This is similar to the mean age of 48.1 years reported by Chinawa and Chime among patients attending the Eye clinic of a faith-based hospital in Abak, south eastern Nigeria.^13^ However, the mean age in this study is higher than the mean age of 41.1 years reported by Ezegwuli et al among patients seen at the Eye clinic of the University of Nigeria Teaching Hospital Enugu, south eastern Nigeria.^2^ Also, the mean age in this study is higher than the mean age of 37.0 years reported by Ogunfowora and Mora among patients attending the general out-patient clinic of a Teaching hospital in Abuja, north central Nigeria.^11^ Eye diseases such as cataract, age-related macular degeneration (AMD), diabetic retinopathy etc are commoner in older population groups.^18^ In addition, authors have reported higher mean ages among patients attending out-patient Ophthalmology clinics 2, 13 compared to those attending general out-patient clinics across Nigeria.^11^, ^12^

Patients spends an average of 180.3 minutes waiting for services. This is higher than the average waiting time of 43.0 ± 38.0 minutes reported in the out-patient Eye clinic of University of Virginia Health System.^19^ However, the additional time spent waiting for other services after seeing the physician was not included in calculating the waiting time variable unlike in this study where the average waiting time variable included waiting times for other services (e.g visual acuity measurement, dilatation etc).

Comparing the average total waiting time in this study with the average total waiting time in the general outpatient clinic (GOPD) of other tertiary hospitals in Nigeria showed the average waiting time in our clinic is long. An average waiting time of 83.7±38.6 minutes was reported in the GOPD of Usman Danfodyio University Teaching Hospital, Sokoto, north-west Nigeria.^5^

A similarly lower average waiting time of 97.2 minutes was reported at the GOPD of the Ahmadu Bello University Teaching Hospital (ABUTH), Zaria, north-west Nigeria.^7^ A median waiting time of 1 hour was found at the National Hospital, Abuja, north-central Nigeria.^11^

The difference in waiting time experienced by patients in this study compared to other general outpatient clinic in the country may be due to differences in the study settings. This study was done at the out-patient general Ophthalmology clinic, which is a medical specialty clinic where patients are examined in greater detail and have to wait to access care from several care givers compared to the general out-patient clinics in a tertiary hospital where patients are basically triaged/sorted into various medical specialties and referred appropriately.

In other African countries, a similarly high mean waiting time as found in this study was reported. A mean waiting time of 149.2±72.1 minutes was recorded in Ethiopia; and in Trinidad and Tobago, a mean waiting time of 160 minutes was recorded.^21^

The long waiting time for services discovered in this study needs to be properly addressed as the waiting time for services is an important indicator of the quality of services offered by the hospital.^12^ More so in Nigeria, where members of the public perceive services provided at public hospitals to be very poor.^12^ More importantly, long waiting time represent an opportunity cost for patients,^15^ as it translates to loss of income to the patients and their relatives. This could affect compliance with follow-up appointment and treatment schedule and ultimately, treatment outcome as patients and their relatives considers the opportunity cost for them.

Patients waited the longest before seeing the doctor. Imbalance in doctor – patient ratio has been cited as a major challenge in several hospitals in Africa including Nigeria.^20^, ^5^ Adepoju and colleagues in a study evaluating the human resource development for Vision 2020 in developing countries reported adequate number of eye care workers in Kwara state, Nigeria, but the challenge is in the staff mix and gaps in cadres.^4^ The ratio of Resident doctors (trainee Ophthalmologists) to Consultant Ophthalmologists is low at UITH. At UITH, Resident doctors perform the initial consultation then, the supervising Consultants outline the final management plan for the patients. This can make patients wait longer on the queue when there are no adequate Resident doctors. Also, Nigeria is presently facing a brain drain problem with several young doctors and healthcare personnel leaving the country in large numbers thereby depleting the available pool of Resident doctors to serve the Eye care facilities. Service time for pupil dilatation and refraction contributed majorly to the bulk of time spent by the patients when accessing service in the clinic. In particular, pupil dilatation. Adequate pupillary dilatation (≥6mm) is expected 40 minutes after instillation of the combination of Tropicamide (0.8%) and Phenylephrine (5%).^21^ this is the mydriatic solution that is used routinely for out-patient funduscopic examination in adults in the ophthalmology clinic at UITH.

Pupil dilatation taking up to an average of 64.0 minutes as found in this study is long. Possible explanation could be that the effect of reflex tearing that occurs when the dilating drops were instilled washed out the drugs thus reducing its efficacy. Also, the study population are of African race with abundant iris pigmentation making their pupils to dilate slowly in response to mydriatic drugs.^21^

The average clinic time spent by patients when they seek Eye care at the out-patient general Ophthalmology clinic of UITH is 240.0 minutes. This is way in excess of the 60 minutes proposed by Mahmoud as the reasonable time a patient is expected to conclude the entire consultation process at the emergency Eye care unit of the National Eye Centre Kaduna, north west Nigeria.^10^ Although the time spent in the Eye clinic by patients varies based on individual case complexities, and thus maybe difficult to establish the ideal clinic time.

Long total clinic time is common in out-patient clinics of developing countries.^11^,^5^,^20^ The total clinic encounter time that lasted up to 7.2 hours was reported in the National Hospital, Abuja, north-central Nigeria.^11^ Total clinic waiting time (from entry to departure) ranging from 1.15 – 4.1 hours was reported in a tertiary hospital in Sokoto, north-west Nigeria.^5^

Patient category and arrival time to the clinic were the factors that are significantly associated with total clinic time among out-patients. Patients coming to the clinic for the first time (new patients) have longer total clinic time compared to follow-up patients (p= 0.01). New patients are not familiar with clinic protocol and may take longer time navigating through the service points. Also, new patients will be clerked, all relevant base line information gotten and documented in their case note for the first time.

The documentation process may take considerable service time unlike follow-up patients where base line information is already documented so clinic contact time is relatively shortened.

Clinic arrival time of patients has a negative relationship (r=-0.153) with the total clinic time with early arriving patients spending longer clinic time. Patients who arrived before 8am to the clinic spent longer clinic time compared with those who arrived much later (p=0.02). Patients arrive clinic early on the assumption that they would receive treatment early and be able to reduce clinic time. This was however not so in this study possibly because majority of patients arrive the clinic before 8 am (figure 1) and given the fixed capacity of the out-patient consulting rooms and staff strength, there is a cluster of patients at the beginning of morning duty by the early arriving patients leading to clinic congestion. So, early arriving patients will have to wait a considerable time for services adding to the total clinic time.

The findings from this study corresponds with studies conducted in Saudi Arabia, Vietnam and China where paradoxically those that registered early waited the longest at the clinics.^16^,^22^ The Ophthalmology clinic of UITH operates an appointment-based system for follow-up patients but new patients are seen on walk-in basis with no strict timeline. This creates variability in the number of patients to be seen per day, and the clinic arrival time of patients. This variability makes planning for services and work scheduling very difficult.

Studies have demonstrated that satisfaction with the time spent waiting was most strongly correlated with overall satisfaction in the out-patient Eye clinic.^19^ Majority of the patients sampled in this study expressed satisfied with the total time spent in the clinic despite expressing that they spent a long time in the clinic. The feeling of satisfaction expressed by the patients may be related to the quality of treatment they received at the clinic making them to overlook the shortcoming in terms of the long time spent at the clinic. A study done in the same department revealed similar findings where the patients reported long waiting time particularly for doctors' consultation but were satisfied with the quality of care they received.^3^ Patients satisfaction with the time spent in the clinic despite waiting a long time was also reported in the Eye clinic of University of Nigeria Teaching Hospital, Enugu, south-east Nigeria.^2^ The presence of sources of entertainment like television and reading materials in patients waiting room have been reported to make patients' wait in the clinic more bearable, and thus could contribute to the expressed patients satisfaction with the clinic time.^2^ Patients are also more likely to express satisfaction with clinic time when they perceive that they had adequate physician contact time. 19, 11 Physician – patient contact time of more than 15 minutes have been found by Christopher et al to be associated with less dissatisfaction with waiting time.^23^

The mean doctor consultation time of 29.0 ±27.5 minutes found in this study is higher than the 9 minutes face-toface physician encounter reported in Abuja, Nigeria.^11^ It is also higher than the 15 minutes proposed by Mahmoud as the maximum time for primary Eye care doctor consultation.^10^ This could possibly be perceived by the patients as adequate physician contact time resulting in the expressed feeling of satisfaction with the clinic time.

The patients as end users of hospital services can provide valuable feedbacks on how to give them a good experience in our clinics. Provision of steady power supply will make services run uninterruptedly, and patients can be discharged from the clinics as soon possible. Re-organising the clinic structure has been suggested by patients as a way to improve clinic experience. Becoming better organised and implementing simple changes such as sign-post and directional sign has been advocated as cheap and effective methods that allows better use of available clinic space and infrastructure that often delay services.^9^ Increasing the number of Eye care personnel and equipment have also been recommended as ways to reduce patient waiting time in clinics,^5^, ^20^ similar to what was suggested by patients in this study. Equipment procurement and employing more personnel to serve the Eye clinic are capital intensive projects. Allocations to the health sector at the Federal level, relative to the budget size, continue to decline, from 5.97% in 2012, to 3.95% in 2018.^24^ The dwindling funding of healthcare by the Federal Government of Nigeria could further worsen the long clinic times if action is not taken to reverse the trend, and make more funds available for health care spending.

Furthermore, improved professionalism of Eye care workers such as early resumption, and increased time efficiency by workers have been demonstrated to be equally important in improving patients waiting time in the clinics especially in situations where resources are limited.^9^ Adepoju et al reported that the main challenge to efficient eye care delivery is with staff distribution and motivation and once these challenges are addressed, output will increase.^4^

### Limitations

This study did not assess the health personnel's contribution to the patient's waiting, service and total clinic time. The work resumption time, hours on break and the number of patients attended to by individual doctors per day which may affect patients' clinic time. This will require a more discrete investigation which is beyond the scope of this study.

## Conclusion

The waiting and service times at the out-patient general Ophthalmology clinic of UITH is long. Patients that had pupil dilatation spend the longest time accessing service. Majority of the patients expressed satisfaction with their clinic time possibly from their long contact with physicians. However, the patients' clinic experience can be improved by providing steady power supply and strictly adhering to the first come first served rule

## Figures and Tables

**Figure 2:**
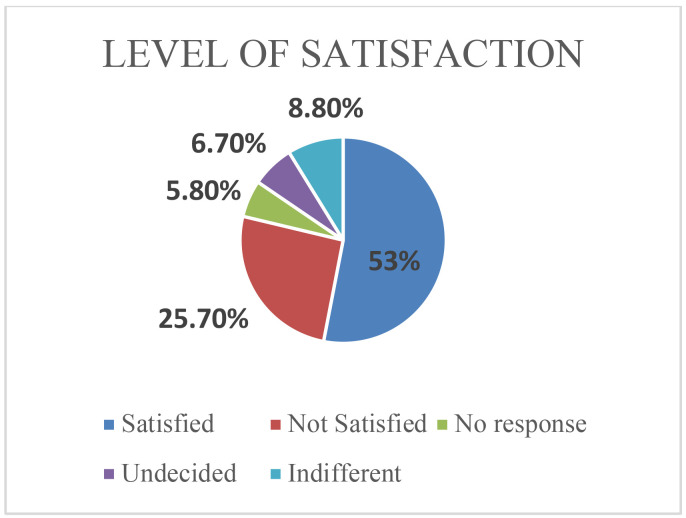
Patients' satisfaction with total time spent in clinic
